# Symptomatic response to CPAP in obstructive sleep apnea versus COPD- obstructive sleep apnea overlap syndrome: Insights from a large national registry

**DOI:** 10.1371/journal.pone.0256230

**Published:** 2021-08-12

**Authors:** Dan Adler, Sébastien Bailly, Paola Marina Soccal, Jean-Paul Janssens, Marc Sapène, Yves Grillet, Bruno Stach, Renaud Tamisier, Jean-Louis Pépin

**Affiliations:** 1 Division of Pneumology, Geneva University Hospitals, Geneva, Switzerland; 2 University of Geneva Faculty of Medicine, Geneva, Switzerland; 3 HP2 Laboratory, INSERM U1042, University Grenoble Alpes, Grenoble, France; 4 EFCR Laboratory, Pole Thorax et Vaisseaux, Grenoble Alpes University Hospital, Grenoble, France; 5 Private Practice Sleep and Respiratory Disease Center, Nouvelle Clinique Bel Air, Bordeaux, France; 6 Private Practice Sleep and Respiratory Disease Center, Valence, France; 7 Private Practice Sleep and Respiratory Disease Center, Valenciennes, France; Harper University Hospital, UNITED STATES

## Abstract

**Background:**

The symptomatic response to continuous positive airway pressure (CPAP) therapy in COPD-obstructive sleep apnea overlap syndrome (OVS) compared to OSA syndrome (OSA) alone has not been well studied so far. The aim of this study is to explore main differences in the clinical response to CPAP treatment in OVS compared to OSA alone.

**Study design and methods:**

Using prospective data from the French National Sleep Apnea Registry, we conducted an observational study among 6320 patients with moderate-to-severe OSA, available spirometry, and at least one follow-up visit under CPAP therapy.

**Results:**

CPAP efficacy measured on the residual apnea-hypopnea index and median adherence were similar between OVS and OSA patients. In both groups, the overall burden of symptoms related to sleep apnea improved with CPAP treatment. In a multivariable model adjusted for age, gender, body mass index, adherence to treatment and residual apnea-hypopnea index, OVS was associated with higher odds for persistent morning headaches (OR: 1.37 [95% CI; 1.04; 1.79]; P = 0.02), morning tiredness (OR: 1.33 [95% CI: 1.12; 1.59]; P<0.01), daytime sleepiness (OR; 1.24 [95% CI: 1.4; 1.46]: P<0.01) and exertional dyspnea (OR: 1.26 [95% CI: 1.00;1.58]; P = 0.04) when compared with OSA alone.

**Interpretation:**

CPAP therapy was effective in normalizing the apnea-hypopnea index and significantly improved OSA-related symptoms, regardless of COPD status. CPAP should be offered to patients with OVS on a trial basis as a significant improvement in OSA-related symptoms can be expected, although the range of response may be less dramatic than in OSA alone.

## Introduction

Chronic obstructive pulmonary disease (COPD) and obstructive sleep apnea (OSA) are among the most prevalent non-communicable chronic diseases and represent major societal problems with an associated economic burden challenging health systems worldwide [1–3]. The association of COPD and OSA is known as the COPD-OSA “overlap” syndrome (OVS) and acknowledged to be linked with a poor prognosis. Although the precise estimate of the prevalence of OVS remains a subject of debate, approximately one out of 10 patients with one disease will have the other disorder by chance alone [4, 5]. OVS represents a specific phenotype in which symptoms related to OSA are distinct and often overlooked [6], resulting in the under-diagnosis of a potentially actionable and curable important comorbid condition, even in the most severe COPD patients [7, 8]. Moreover, air trapping and static hyperinflation may contribute to low arousal threshold and high loop gain in OVS with its associated ventilatory instability [9, 10].

Continuous positive airway pressure (CPAP) is the first-line therapy for moderate-to-severe OSA [11]. Symptomatic responses to CPAP have been well established in randomized controlled trials and have consistently reported a significant improvement in excessive daytime sleepiness, quality of life and work productivity [12]. Treatment is so effective on neurocognitive outcomes that it is no longer considered ethical to design long-term randomized trials on cardiovascular outcomes including sleepy patients. The range of the symptomatic response to CPAP is highly dependent on OSA clinical phenotypes [13–15], but it remains significant, even in minimally symptomatic individuals who represent 50% of patients at initial presentation [16, 17]. Historically, CPAP was also recommended to reduce cardiovascular and metabolic risks associated with OSA, but there is currently no high-quality evidence from randomized controlled trials supporting this indication [17, 18]. Consequently, interventions such as CPAP are now essentially recommended in symptomatic patients with expected immediate individual benefits.

We have previously demonstrated that a lower proportion of OVS patients with coexisting OSA complained of snoring, morning headaches and excessive daytime sleepiness, as well as experiencing sleepiness while driving [6]. However, data regarding the symptomatic effects of CPAP in the specific OVS population are lacking. We investigated the respective clinical responses to CPAP treatment in OVS compared to OSA alone among adults included in the French National Sleep Apnea Registry.

## Methods

### Study design and population

This observational study was conducted using prospectively collected data from the French National Sleep Apnea Registry (Observatoire Sommeil de la Fédération de Pneumologie [OSFP; www.osfp.fr]). The OSFP registry is a standardized web-based report administered by the French Federation of Pulmonology. It comprises standardized, anonymized, longitudinal data of patients over 18 years old referred for suspected sleep-disordered breathing, assessed by respiratory physicians working in private practice and general or university hospitals. In France, a one-year visit is mandatory for the renewal of the reimbursement of CPAP devices by the public national health system. This visit should include documentation of symptoms evolution and objective data on CPAP adherence. Participating staff are trained in the use of electronic data format with the appropriate software. Periodic quality control checks are performed to ensure up-to-standard data recording and to ensure a low rate of missing data.

In the current study, 51,450 patients registered in the database between January 1997 and January 2017 with an apnea-hypopnea index (AHI) ≥15 and/or oxygen desaturation index ≥15 were included for analysis. Valid measurement of forced expiratory volume in 1 second (FEV1) over forced vital capacity (FVC) was available for 18,521 patients. Among these, 6,320 patients had at least one follow-up visit under CPAP and were included in the final analysis. In the registry, demographic data and medical history are collected during baseline evaluations, including diagnosis of cardiovascular comorbidities validated by a physician. Symptoms are evaluated using standardized questionnaires including Epworth Sleepiness Scale (ESS), Pichot fatigue scale, Pichot Depression scale and main OSAS symptoms at baseline and at follow-up visits to assess treatment efficacy.

Ethics committee approval to set up the study database was obtained from the French information technology and personal data protection authorities (C.C.T. I.R.S no. 09.521). The OSFP independent scientific advisory committee approved the use of this database for the present study. All patients included gave written informed consent.

### Sleep studies

Full night, in-laboratory, attended polysomnography or type III cardiopulmonary sleep recordings are required by the OSFP registry for OSA diagnosis. In France, sleep stages and arousals are usually scored using the American Academy of Sleep Medicine criteria [19]. Apnea is scored if a drop of 90% or more in airflow signal excursion is noted for at least 10 seconds. Hypopnea is defined as a drop greater than or equal to 30% in airflow lasting at least 10 seconds and associated with 3% oxygen desaturation or electroencephalogram arousal. Type III cardiopulmonary sleep recordings are a reliable, cost-effective option for suspected OSA [20]. Interpretation of type III recording using hypopnea criteria, which includes only a 30% drop in airflow and 3% desaturation, has been demonstrated to have the best diagnosis accuracy compared to full polysomnography [21]. AHI cut-off values between 15 and <30 were used to classify moderate sleep apnea and a cut-off value ≥30 was used for severe sleep apnea for polysomnography and type III cardiopulmonary sleep recording. Data of sleep architecture and sleep stages are not available in the database but various indices of sleep apnea severity are collected: Apnea+ Hypopnea index (AHI), mean and minimal nocturnal SaO2 and time spent at SaO2 below 90%.

### Spirometry

Pulmonary function tests are recommended in France as part of the initial workup for OSA in the case of current or past cigarette smoking and/or obesity (body mass index [BMI] >30 kg/m2) and/or respiratory symptoms, such as dyspnea [22]. A post-bronchodilator fixed FEV1/FVC ratio <70% is used to define COPD. Specific spirometric FEV1 (percentage of predicted) cut points were used to assess airflow limitation severity: 1) GOLD 1 (FEV1 ≥80%); 2) GOLD 2 (50% ≤FEV1 <80%); 3) GOLD 3 (30% ≤FEV1 <50%); 4) GOLD 4 (FEV1<30%).

### Statistical analysis

The description of quantitative variables was performed using the median and interquartile range (IQR) and the number and percentage were used to describe qualitative variables. Comparisons between groups (OSA versus OVS) were performed using a non-parametric Mann-Whitney test for continuous variables and a Chi-square test for qualitative variables. To determine the factor associated with the symptomatic response, multiples logistic regression models were performed for all symptoms related to OSA as a dependent variable and adjusted for age, gender, BMI, residual AHI and CPAP adherence after verification for the absence of collinearity. As log linearity was not verified for age and BMI, these variables were transformed into categorical variables (for age: <56 years old, [56, 65], [65, 72] and greater than 72 years old; for BMI: <25 normal, [25–30] overweight, [30–35] obese and > = 35 extremely obese). Missing values were observed for age and CPAP adherence and thus multiple imputations were performed to account for these. Ten datasets were constituted. No significant modifications were observed in the imputed dataset for this variable compared to the non-imputed dataset. Statistical analyses were performed using SAS V.9.4 (SAS Institute Inc., Cary, NC). A P value threshold of 0.05 was considered as significant.

## Results

### Study population

Of the 6,320 patients with a diagnosis of OSA and available spirometry included in this study, 5,566 (88.1%) had OSA only and 754 (11.9%) were diagnosed with OVS. Patient demographics, pulmonary function tests, sleep studies and comorbidities at the time of inclusion in the Registry are presented in Table 1. OVS patients were more often older males with a higher exposure to tobacco smoking compared to OSA patients. Both OVS and OSA patients presented a high burden of prevalent cardiovascular and metabolic comorbidities.

**Table 1:** Patient demographics, pulmonary function tests, sleep studies and comorbidities at inclusion.

Variables	Items	SAS	Overlap	Missing	P-value
		N = 5,566 (88.1%)	N = 754 (11.9%)		
**Anthropometrics**					
Gender (%)	Male	4,022 (72.5)	616 (81.8)	17	<0.01
Age (years, median?)		57.4 [49; 65.1]	64.1 [56.6; 71.4]	0	<0.01
BMI (kg/m^2^)		31.6 [27.8; 36.1]	31.6 [27.9; 36.6]	64	0.89
Alcohol use (%)	Yes	238 (4.3)	78 (10.4)	50	<0.01
Smoking status (%)	non-smoker	2,788 (50.3)	229 (30.5)	23	<0.01
	former smoker	1,857 (33.5)	359 (47.8)	.	.
	current smoker	901 (16.2)	163 (21.7)	.	.
Sedentary	Yes	1,327 (24)	208 (27.8)	39	0.02
**COPD status**					
COPD Gold stage	No	5,566(100)	0		
	GOLD stage 1	0	319 (42.76)		
	GOLD stage 2	0	344 (46.11)		
	GOLD stages 3 &4	0	83 (11.13)		
Forced expiratory volume in one second (FEV1) (%)		97 [85; 107]	75 [61; 90]	170	<0.01
FEV1/SVC (%)		82 [77; 87]	64 [59; 68]	0	<0.01
**Sleep studies**					
Type of sleep studies (%)	Polysomnography	2,308 (42.8)	331 (45.6)	205	0.16
	Polygraphy	3,081 (57.2)	395 (54.4)	.	.
Apnea + hypopnea index		39 [30; 56.4]	41 [32; 59]	9	<0.01
Oxygen desaturation index (event/hour)		31 [17.6; 51]	32.3 [17; 52]	686	0.85
Mean nocturnal Sa02		92.8 [91; 94]	92 [90; 93]	1229	<0.01
Time spent with SaO2<90% (min)		34[8; 106]	54[16; 163]	2550	<0.01
**Presence of comorbidities**					
Stroke (%)		165 (3)	33 (4.4)	33	0.04
Arteriopathy (%)		120 (2.2)	42 (5.6)	36	<0.01
Diabetes (%)		1,086 (19.5)	169 (22.4)	0	0.06
Hypertension (%)		2,589 (46.7)	432 (57.8)	33	<0.01
Hypertriglyceridemia (%)		412 (7.6)	60 (8.3)	172	0.49
Heart failure (%)		110 (2)	29 (3.9)	33	<0.01
Coronary artery disease (%)		330 (6)	83 (11.1)	33	<0.01
Myocardial infarction (%)		201 (3.6)	59 (7.9)	24	<0.01

### Symptoms at baseline

The landscape of symptoms differed between groups at baseline (Table 2A). Morning headaches (39.1% versus 32.9%; P<0.01), sleepiness while driving (46.8% versus 41.5%; P<0.01) with near- miss road accidents (14.1% versus 10.8%; P<0.04), and self-reported attentional disorders (18.4% versus 10.2%; P<0.01) were more prevalent in OSA patients compared to the OVS population. Conversely, a lower proportion of OSA patients complained of nocturia (63.6% versus 72.9%; P<0.01) and erectile dysfunction (25.0% versus 30.9%; P<0.01) (Table 2A).

**Table 2:** Between-group difference in symptoms at baseline and follow-up visit.

Variables	OSA	OVS	Missing	P-value
	N = 5,566 (88.1%)	N = 754 (11.9%)		
**A. Baseline**				
Morning headaches, n(%)	2,176 (39.1)	248 (32.9)	0	< .01
Exertional dyspnea, n(%)	3,110 (55.9)	437 (58)	0	0.28
Depression scale, median [IQR]	3 [1; 7]	3 [1; 7]	1004	0.81
Epworth Sleepiness Scale, median [IQR]	10 [7; 14]	10 [6; 14]	358	0.12
Pichot’s fatigue scale, median [IQR]	14 [7; 20]	13 [6; 20]	913	0.21
Morning tiredness, median [IQR]	4,330 (77.8)	597 (79.2)	0	0.39
Nocturia, n(%)	3,523 (63.6)	547 (72.9)	27	< .01
Near miss accidents, n(%)	639 (14.1)	61 (10.8)	1210	0.04
Sleepiness at the wheel, n(%)	2,605 (46.8)	313 (41.5)	0	< .01
Daytime sleepiness, n(%)	4,796 (86.2)	668 (88.6)	0	0.07
Night sweating, n(%)	2,538 (45.6)	340 (45.1)	0	0.79
Erectile dysfunction, n(%)	1393 (25)	233 (30.9)	0	< .01
Attentional disorders, n(%)	1,023 (18.4)	77 (10.2)	0	< .01
**B. Follow-up visit**				
Morning headaches, n(%)	1,184 (21.3)	173 (22.9)	0	0.29
Exertional dyspnea, n(%)	2,256 (40.5)	339 (45)	0	0.02
Depression scale, median [IQR]	1 [0; 4]	1 [0; 5]	1811	0.05
Epworth Sleepiness Scale, median [IQR]	6 [3; 9]	5 [3; 8]	1299	0.07
Pichot’s fatigue scale, median [IQR]	6 [2; 12]	7 [2; 13]	1681	0.20
Morning tiredness, median [IQR]	2,395 (43)	376 (49.9)	0	< .01
Nocturia, n(%)	2,185 (39.7)	389 (52.4)	73	< .01
Near-miss accidents, n(%)	226 (5)	26 (4.7)	1214	0.76
Sleepiness at the wheel, n(%)	902 (16.2)	124 (16.4)	0	0.87
Daytime sleepiness, n(%)	2,683 (48.2)	401 (53.2)	0	0.01
Night sweating, n(%)	1,496 (26.9)	217 (28.8)	0	0.27
Erectile dysfunction, n(%)	1,006 (18.1)	167 (22.1)	0	< .01
Attentional disorders, n(%)	574 (10.3)	44 (5.8)	0	< .01

A CPAP therapy follow-up visit was reported after a median of 517 days (IQR: 216; 616) for OSA patients and 536 days (IQR: 326; 729) for OVS patients. Median adherence to CPAP was similar (6 h [IQR: 4.9; 7 and IQR: 5; 7]) for both OSA and OVS patients, respectively (P = 0.34). CPAP efficacy was also similar between the two groups with a median residual AHI of 2.7/h (IQR: 1.3; 5) and 3.0/h (IQR: 1.5; 5.1) for OSA and OVS patients, respectively.

### Symptomatic responses to CPAP treatment

The overall burden of symptoms related to sleep apnea improved with CPAP treatment in both groups. At the follow-up visit, a descriptive analysis showed no difference in the proportion of persistent morning headaches, sleepiness at the wheel, near-miss road accidents, and self- reported attentional disorders between OSA and OVS patients under CPAP therapy. However, there was a higher baseline prevalence of nocturia and erectile dysfunction in OVS patients that appeared to be resistant to CPAP treatment (Table 2B).

**Fig 1:**
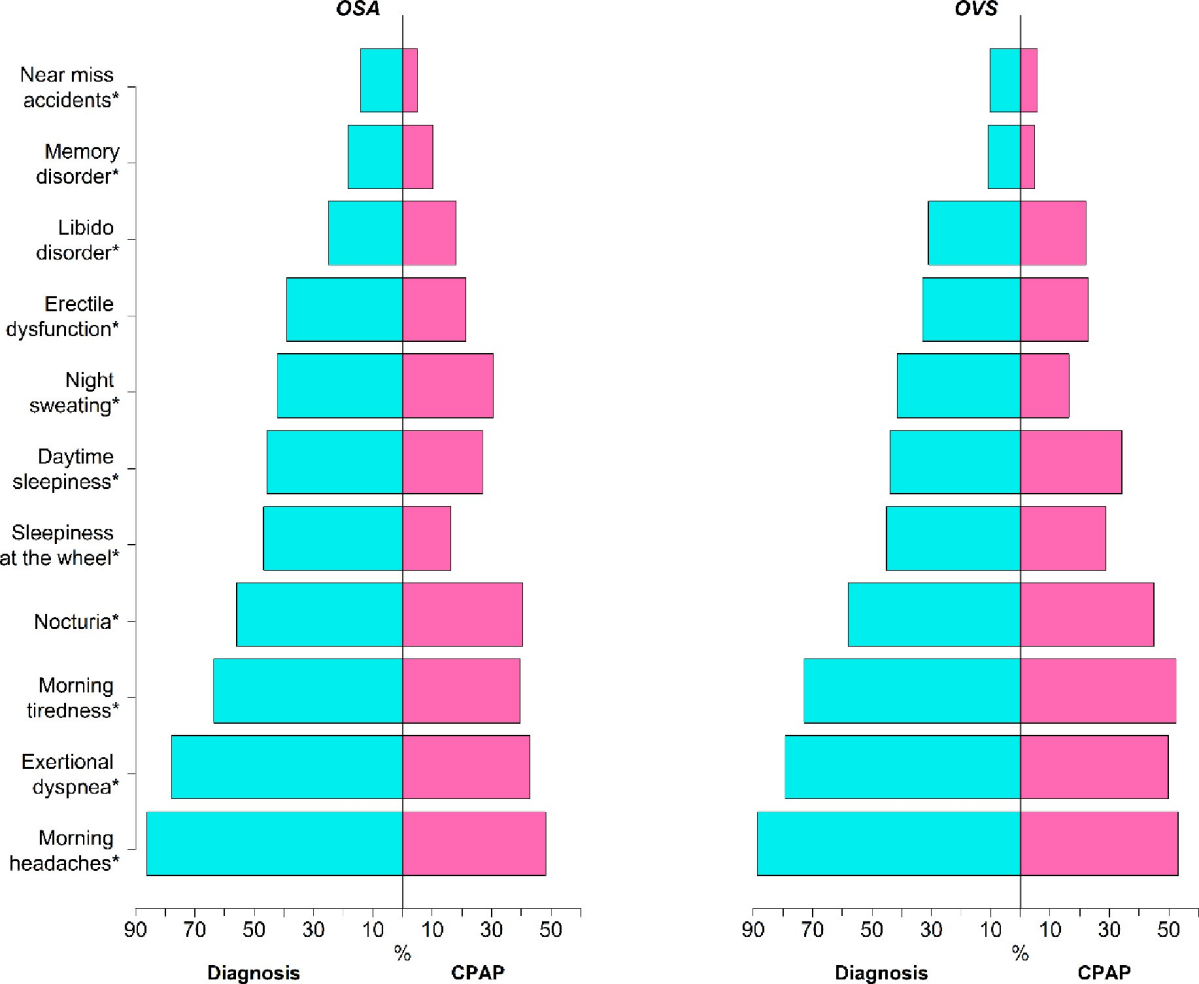
Proportion of symptoms at baseline (blue) and follow-up visit (pink) in OSA patients (left) and OVS patients (right). * denotes a significant difference between baseline and follow-up visit.

**Fig 2:**
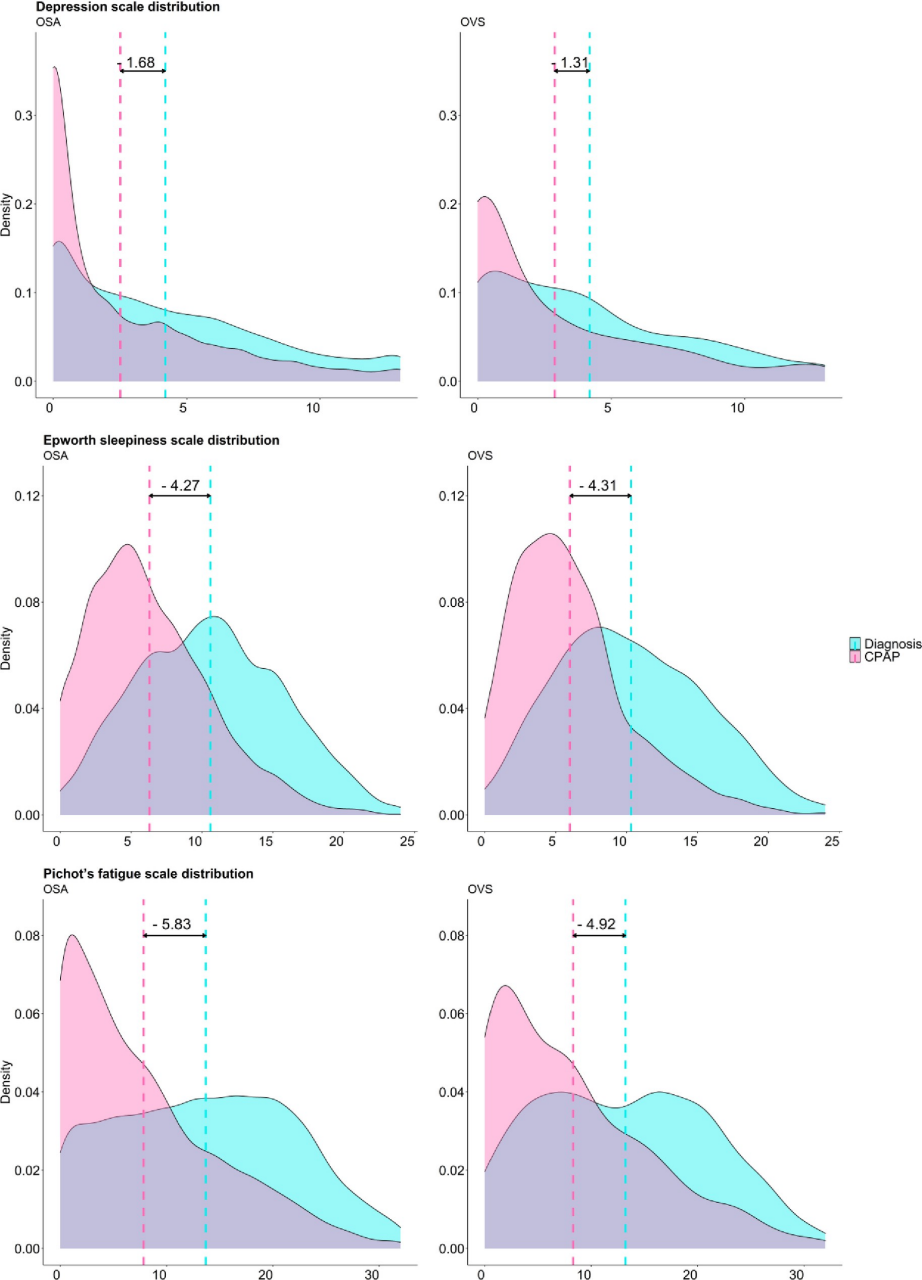
Distribution of Depression scale, ESS and Pichot’s fatigue scale at baseline (blue) and follow-up visit (pink) in OSA patients (left) and OVS patients (right). The scattered line represents median value. The difference between both scattered lines represent the effect of CPAP treatment.

**Fig 3:**
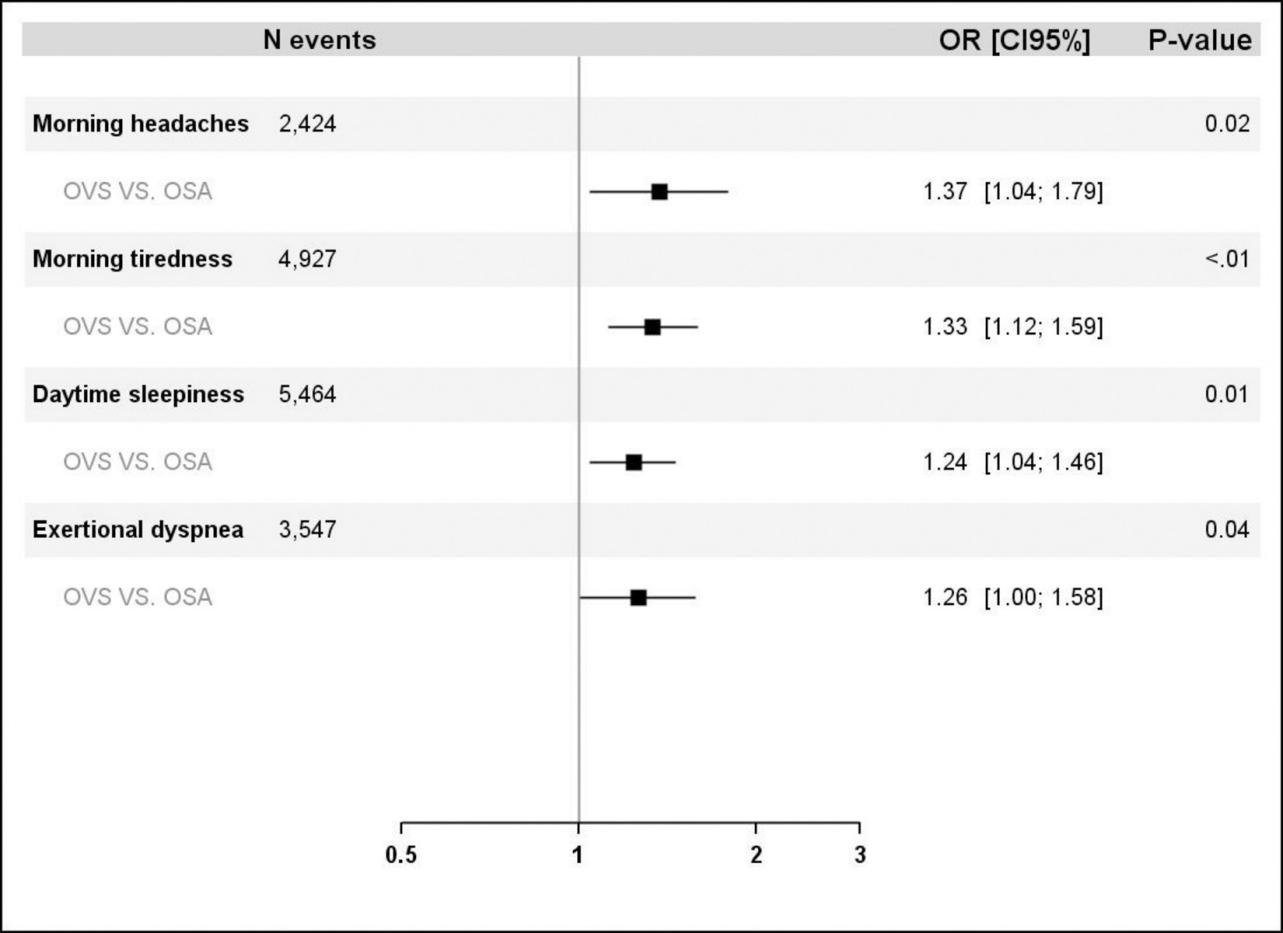
Association between OVS and morning headaches, morning tiredness, daytime sleepiness and exertional dyspnea after controlling for age, gender, BMI, adherence to treatment and residual AHI.

A significant proportion of patients in both groups had improved OSA-related symptoms (Fig 1). CPAP treatment also had a major favorable effect on specific scales for daytime excessive sleepiness (Epworth Sleepiness Scale [ESS]), daytime fatigue (Pichot scale) and depression (Pichot scale) in both groups (Fig 2). In a multivariable model adjusted for age, gender, BMI, adherence to treatment and residual AHI, OVS was associated with higher odds for persistent morning headaches (OR: 1.37 [95% CI: 1.04; 1.79]; P = 0.02), morning tiredness (OR: 1.33 [95% CI: 1.12; 1.59]; P<0.01), daytime sleepiness (OR: 1.24 [95% CI: 1.4; 1.46]; P<0.01) and exertional dyspnea (OR: 1.26 [95% CI: 1.00;1.58]; P = 0.04) (Fig 3) compared to OSA.

## Discussion

In this large real-life sleep apnea registry which also included mild and moderate COPD patients, we observed that CPAP therapy was effective in normalizing AHI and significantly improved OSA-related symptoms, namely sleepiness and fatigue, regardless of COPD status. As expected, some symptoms more prevalent in COPD patients at treatment initiation were less responsive to CPAP therapy, such as dyspnea, nocturia and erectile dysfunction. A multivariable model accounting for main confounders indicated that the range of responses was lower in the OVS group.

Results of this registry cohort show that the median improvement in the ESS with CPAP therapy was -4.31 points (95% CI: -4.79; -3.83) in the OVS group, which is far beyond the currently established 2 points minimal clinically important difference [23]. Our findings are consistent with data from recent randomized trials of CPAP therapy in minimally symptomatic OSA populations at high cardiovascular risk. The Sleep Apnea CardioVascular Endpoints (SAVE) study randomized 2,717 moderate-to-severe non-sleepy patients with cardiovascular or cerebrovascular disease to CPAP therapy or usual care. Over a mean follow-up of 3.7 years, no difference was observed between groups in the occurrence of the composite primary endpoint of myocardial infarction, stroke, cardiovascular death, or hospitalization for heart failure, acute coronary syndrome, or transient ischemic attack. However, CPAP therapy significantly improved OSA-related symptoms, health-related quality of life and mood. For example, the treatment group demonstrated a mean improvement in the ESS of -3.1±4.1 points [17].

The Impact of Sleep Apnea in the Evolution of Acute Coronary Syndrome (ISAAC) study randomized 1,264 non-sleepy patients with an acute coronary syndrome and moderate-to- severe OSA to CPAP therapy or usual care. Over a median follow-up of 3.4 years, no difference in the occurrence of the composite primary endpoint of a recurrent cardiovascular event was observed between treatment groups. However, CPAP therapy was still associated with decreased daytime sleepiness as measured by ESS, although clinically less important than in the SAVE study [24]. The Multi-centre Obstructive Sleep Apnoea Interventional Cardiovascular (MOSAIC) trial was specifically designed to identify a symptomatic benefit of CPAP therapy in minimally symptomatic patients. At baseline, ESS was 8.0±4.2 among 196 patients randomized to usual care and 7.9±4.4 among 195 patients randomized to CPAP therapy. At 6 months, the adjusted treatment effect with CPAP therapy was ESS -2.1 (95% CI: -2.6 to -1.4; P<0.0001) [16].

Despite an overall improvement of symptoms, higher odds for persistent morning headaches, morning tiredness, daytime sleepiness and exertional dyspnea were found in OVS patients. Of note, this is not contradictory with data derived from crude comparisons between groups. The “take home message” for clinicians is that the OVS population is indicated for CPAP therapy, but there is more variability in the range of response related to cormorbid COPD compared to OSA alone and this might lead to disappointment in some OVS patients. Indeed, there are no clear ways to predict in advance which OVS will benefit from CPAP therapy. Our results suggests that some OSA phenotypes such as minimally symptomatic OVS patients still benefit from CPAP therapy to improve OSA-related symptoms that may have been attributed to COPD. Our findings may be of particular interest for OVS patients, who often represent a “silent population” of unrecognized and undertreated comorbid OSA [7, 8, 25].

Diagnosis of OSA in COPD patients remains a real clinical challenge as classical predictors of sleep apnea, such as age, gender, neck circumference and the ESS, do not perform well in this less symptomatic population [6, 26, 27]. COPD and OSA share intermediate pathophysiological mechanisms that are linked to elevated blood pressure, systemic inflammation, metabolic dysregulation and endothelial dysfunction [28]. We acknowledge that recent randomized trials have failed to demonstrate any benefit of CPAP for the secondary prevention of cardiovascular events in classical OSA. However, as OVS patients derived a symptomatic benefit from CPAP therapy in our study and given that COPD is a recognized cardiovascular risk factor [29, 30], more studies addressing the effect of CPAP therapy on cardiovascular health in OVS should be designed [31].

Our study has several strengths, notably robust, prospectively collected data from a national registry. We report here symptomatic responses to CPAP therapy in the largest OVS population to date and for which both OSA and COPD severity were objectively characterized. Our data suggest a significant symptomatic impact of CPAP therapy in the overall OVS population. In a real-life registry using well-maintained electronic medical records, a careful analysis of confounders also allowed to report smaller differences in response to treatment between OSA and OVS groups.

Certain limitations should also be noted. First, only patients with a valid spirometry were included in the present analysis resulting in a selection of an OSA phenotype with more respiratory complaints. However, we assume the magnitude of this potential bias to be low since indications for performing pulmonary function tests in the OSA clinical workflow in France are wide with recommendations of scientific societies to investigate all OSA patients with a BMI > 30 kg/m2 and/or any history of tobacco exposure. Second, as this study was carried out in a sleep clinic, a majority of OVS patients included for the analysis had mild or moderate COPD. Therefore, our results are valid for this setting and benefits related to CPAP treatment might not be generalizable to more advanced OVS patients in GOLD stages 3 and 4. Moreover, only patients with at least one follow-up visit were included in our study, thus potentially corresponding to a selection of a more responsive population of OVS with favorable outcomes. Indeed, recent data suggest that the CPAP termination rate is higher in the association of COPD and OSAS but at one year this difference do not exceed 5% [32]. Therefore, the impact on our results is expected to be limited.

## Conclusions

CPAP therapy is associated with a global improvement of OSA-related symptoms, irrespective of OVS status, but this positive treatment effect is more variable and difficult to anticipate in OVS patients. Building on previous knowledge that OVS can be minimally symptomatic [6], CPAP should be offered to patients with OVS on a trial basis as important improvements in daytime sleepiness, fatigue and neuropsychological symptoms can be expected.

## Abbreviations

AHI: apnea-hypopnea index
BMI: body mass index
CPAP: continuous positive airway pressure
FEV1: forced expiratory volume in 1 second
FVC: forced vital capacity
ISAACC: Impact of Sleep Apnea in the Evolution of Acute Coronary Syndrome (study)
MOSAIC: Multi-centre Obstructive Sleep Apnoea Interventional Cardiovascular (study)
OSA: obstructive sleep apnea
OVS: overlap syndrome
SAVE: Sleep Apnea CardioVascular Endpoints (study)
